# Association between Genetic Polymorphisms in Interleukin Genes and Recurrent Pregnancy Loss – A Systematic Review and Meta-Analysis

**DOI:** 10.1371/journal.pone.0169891

**Published:** 2017-01-19

**Authors:** Meixiang Zhang, Jiawei Xu, Xiao Bao, Wenbin Niu, Linlin Wang, Linqing Du, Nan Zhang, Yingpu Sun

**Affiliations:** Center for Reproductive Medicine, The First Affiliated Hospital of Zhengzhou University, Zhengzhou, People’s Republic of China; University of Sydney, AUSTRALIA

## Abstract

Interleukins are a group of immunomodulatory proteins that mediate a variety of immune reactions in the human body. To investigate the association between interleukin gene polymorphisms and recurrent pregnancy loss (RPL), we reviewed 21 studies from MEDLINE, EMBASE, OVID SP and PubMed to evaluate RPL-related interleukin gene polymorphisms. Meta-analysis was performed on 12 of the polymorphisms, and a review included the others. Our integrated results indicated that IL-1β (-511C/T) (P = 0.02, 95% CI 0.77[0.62,0.96]), IL-6 (-634C/G) (P<0.001, 95% CI 2.91[2.01,4.22]), IL-10 (-1082G/A, –819T/C) (P = 0.01, 95% CI 0.80[0.67,0.96]; P<0.01, 95% CI 0.66[0.49,0.89]), and IL-18 (-137G/C, -105G/A) (P<0.01, 95% CI 1.69[1.24,2.31]; P = <0.01, 95% CI 1.41[1.17,1.70]) consistently associated with RPL after meta-analysis. IL-17A rs2275913 and IL-17F rs763780, IL-21 rs2055979 and rs13143866, IL-1β (-31C/T), IL-6 (-2954G/C), and IL-10 (-536A/G) were reported only once as having a significant association with RPL. The potential mechanism underlying miscarriage and these polymorphisms and future research directions are also discussed.

## Introduction

Recurrent pregnancy loss (RPL), defined as three or more consecutive pregnancy losses before 20 weeks of gestation, affects approximately 1–5% of pregnant women[1]. The etiology of RPL is still unclear. Among the large number of miscarriage-related factors, those involved in abnormal immune reactions and interleukins are striking[2]. Pregnancy depends on the induction of maternal tolerance to fetal tissues; decidual cells will inhibit maternal immunity during pregnancy. Natural killer cells migrating into the uterus during implantation and coordinate the secretion of cytokines that help or limit trophoblast invasion[3]. An increase in of Th2 cells and a decrease in Th1 cells type will protect the allogeneic fetus from infiltrating cytotoxic T cells[3].

Interleukins are a group of immunomodulatory proteins leading a variety of immune reactions in human body. These cytokines also include many molecules that play a part in human conception[2]. IL-1 is produced by cytotrophoblasts at the fetal-maternal interface during early pregnancy and is involved in trophoblast invasion and tissue repair[4]. IL-6, secreted by decidual cell populations, is a potent pro-angiogenic cytokine that stimulates the proliferation of endothelial cells in vitro and regulates the behavior of the female reproductive tract and gestational tissues[5, 6]. IL-10, produced by cytotrophoblasts and decidual T cells, protect the fetal-placental interface by reducing the cytokine secretions of Th1 cells and macrophages[7]. IL-17-positive T cells accumulate in both the decidua and the peripheral blood in patients with RPL[8]. IL-18 induces INF-α, leading to the activation of NK cells, which are involved in uterine vascularization and implantation[9]. IL-21 was also identified as a susceptibility gene for RPL[10]. IL-6, IL-10 and IL-18 plasma concentrations are higher in women with successful pregnancies than in women with RPL[11]. Variants of genes alter the corresponding protein expression levels. Therefore, it was necessary to assess the global frequencies of the variant alleles of interleukins that might cause RPL.

Single-nucleotide polymorphisms (SNPs) are variations at a single nucleotide position in DNA sequence among individuals. If more than one percent of the population carries the different nucleotide at a specific position in the DNA sequence, this variation is defined as a SNP. They can occur in non-coding regions, such as the promoters, and coding regions, such as the gene body at a total frequency of approximately every 200–1000 bases. SNPs in promoters are suspected to affect transcription factor binding[12], which may in turn influence interleukin production and thus be associated with RPL. While SNPs primarily originate as genetic adaptions, genetic recombination and mutations. The biggest difference between SNPs and mutations is that SNPs are inherited. Most SNPs have no effect on development or health. But some of these genetic differences have proven to be very important to human health. They could alter individual response to specific drugs, susceptibility to environmental factors and increase the risk of developing a particular disease. If those inherited SNPs are high-risk candidates, they deserve our attention, and their investigation will lead to further mechanistic research to develop new treatment programs.

## Materials and Methods

### Study selection

We searched the following electronic databases: EMBASE (1980 to March 2015), MEDLINE (1950 to March 2015), OVID SP and PubMed (last update March 2015). We used the terms “recurrent pregnancy loss” (which also included the keywords recurrent miscarriage; spontaneous abortion; termination of pregnancy; fetal loss); “interleukin” (which also included the keywords cytokine; IL); “polymorphism” (which also included the keywords genetic variant; SNP). Various combinations of the terms (e.g., “recurrent miscarriage” AND “IL” AND “polymorphism”) were searched in the databases. Seven genes (IL-1β, IL-4, IL-6, IL-10, IL-17, IL-18 and IL-21) were selected in the first stage, and then we restricted the meta-analysis to the articles on IL-1β, IL-6, IL-10 and IL-18, to the meta-analysis. Relevant articles were obtained by manual search and retrieval. All the authors compared these papers carefully to ensure none of the papers were duplicated in the analysis.

The inclusion criteria were as follows: (i) Case-control study that genotyped patients with RPL and normal controls; (ii) RPL was defined as three or more losses before 20 weeks of pregnancy; (iii) Genotypes were identified by polymerase chain reaction (PCR) or SNP array; and (iv) Polymorphisms information were observed in candidate genes. When faced with overlapping study populations, we generally selected only the study with the most extensive data. If the entire study did not meet the inclusion criteria but one subset did, only the subset was eligible. Studies that did not provide reliable explanations of the selection criteria and the distribution of the genotypes were excluded. Reviews and meta-analyses were excluded.

The following details were retrieved from the included studies: author’s name, country where the study was performed, publication year, study design, definition of RPL, inclusion and exclusion criteria of the RPL and control groups, number of patients in each group, and genotype detection methods. Two authors performed the data extraction independently. In the case of disagreement, a joint review of the study was completed by discussion. The quality was evaluated using the Newcastle–Ottawa scale[13]. Finally, PRISMA 2009 checklist (Preferred Reporting Items for Systematic Reviews and Meta-Analyses: The PRISMA Statement. PLoS Med 6(6): e1000097. doi:10.1371/journal.pmed1000097) and Meta-analysis on Genetic Association Studies Checklist were used for this meta-analysis (S1 and S2 Checklists).

### Statistical analysis

Odds ratios (ORs) and 95% confidence intervals (CIs) from individual studies were calculated for both dominant (AA+AB vs. BB) and recessive (AA vs. AB+BB) genetic models[14] with the fixed effects and random effects models[15, 16]. A P-value of <0.05 was considered significant. Heterogeneity of the exposure effects was statistically assessed with *I*^*2*^[17]. After exploring the source of heterogeneity, we found that it arose from variations in the features of the population, exposure and study quality. RevMan 5 (Cochrane collaboration, Oxford, UK) was used to perform the statistical analyses.

## Results

**Fig 1:**
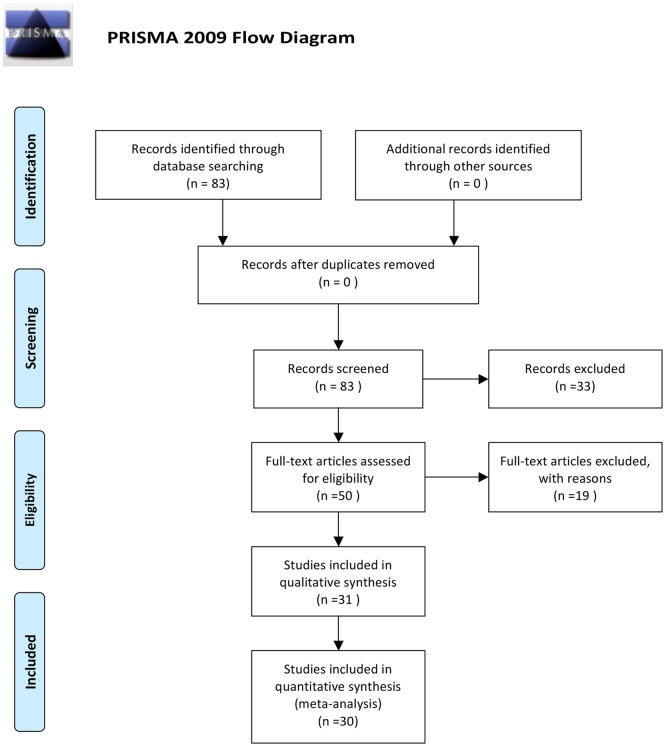
Flow diagram detailing selection of studies for inclusion. *From*: Moher D, Liberati A, Tetzlaff J, Altman DG, The PRISMA Group (2009). *P*referred *R*eporting *I*tems for *S*ystematic Reviews and *M*eta *A*nalyses: The PRISMA Statement. Plos Med 6(7): e1000097. doi: 10.1371/journal.pmed1000097 **For more information, visit** www.prisma-statement.org.

The search strategy yielded 83 citations, and 33 citations were excluded after title and abstract screening. Fifty full manuscripts were obtained for detailed evaluation and scrutiny. Sixteen studies were excluded for the following reasons: five studies were excluded because they were reviews; four studies were excluded for different study objectives; seven studies were excluded because they focused on protein levels; and three studies were excluded because they lacked genotyping data (Fig 1). Ultimately, 31 studies were included (Table 1): 30 articles were used for the meta-analysis, and one article only applied to the literature review because it was the only report of these interleukins[8, 10, 18]. Seven articles assessed IL-1β and included -511C/T, +3593C/T, -1903C/T, -31C/T and -5887C/T[1, 4, 19–23]; twelve articles evaluated IL-6 gene promoter polymorphisms, including -174G/C, -634C/G, -572G/C, -597G/A, -1363G/T, and -2954G/C[3, 5, 14, 24–32]; eleven articles assessed IL-10 gene promoter polymorphisms, including -1082G/A, -592C/A, -819T/C, and -536A/G[3, 14, 23, 24, 30, 31, 33–37]; and five articles evaluated IL-18 gene promoter polymorphisms and included -137G/C, -607C/A, -119A/C, -105G/A, and -656C/A[9, 38–41].

**Table 1:** Methodological characteristics and quality control of all eligible articles.

No.	Author	Year	Origin	Cases	Controls	Alleles studied	Newcastle-Ottawa scale		
				(n)	(n)		Selection	Comparability	Outcome*
1	Karhukorpi J	2001	Finland	38	131	IL-10 (-1082G/A)	****	*	*
2	Hefler LA	2001	Austria	131	68	IL-1β (+3593C/T)	****	*	*
3	Reid JG	2001	UK	40	40	IL-1β (+3954C/T)	***	*	*
4	Babbage SJ	2001	UK	43	73	IL-10 (–1082G/A)	****	*	*
5	Hefler LA	2002	Austria	130	67	IL-1β (-511C/T)	****	*	*
6	Wang ZC	2002	USA	262	144	IL-1β (-511C/T)	****	*	*
						IL-1B (+3953C/T)			
7	Daher S	2003	Brazil	48	108	IL-6 (-174 G/C)	****	*	*
						IL-10 (-1082 G/A)			
8	Unfried G	2003	Austria	161	124	IL-6 (-174G/C)	****	*	*
9	Prigoshin N	2004	Argentina	41	54	IL-6 (-174G/C)	****	*	*
						IL-10 (-1082G/A)			
						IL-10 (-819C/T)			
						IL-10 (-592C/A)			
10	Saijo Y	2004	Japan	76	93	IL-6 (–174G/C)	****	*	*
						IL-6 (–634C/G)			
11	Kamali-Sarvestani E	2005	Iran	139	143	IL-10 (–592A/C)	****	*	*
						IL-10 (–819T/C)			
						IL-10 (–1082 G/A)			
12	Von Linsingen R	2005	Brazil	57	74	IL-6 (–174G/C)	****	*	*
13	Linjawi S	2005	UK	206	224	IL-1β (-511C/T)	****	*	*
14	Naeimi S	2006	Iran	102	103	IL-18 (-137G/C)	****	*	*
						IL-18 (-607C/A)			
15	Zammiti W	2006	Tunisia	350	200	IL-10 (–592C/A)	****	*	*
						IL-10 (–819C/T)			
						IL-10 (–1082A/G)			
16	Ostojic S	2007	Croatia	125	136	IL-18 (-137G/C)	****	*	*
						IL-18 (-607C/A)			
						IL-12B			
17	Al-Khateeb GM	2011	Bahrain	282	289	IL-18 (-119A/C)	****	*	*
						IL-18 (-137G/C)			
						IL-18 (-105G/A)			
						IL-18 (-656C/A)			
18	Kaur A	2011	India	50	50	IL-10 (-592C/A)	****	*	*
19	Messaoudi S	2011	Tunisia	235	235	IL-21	****	*	*
20	Agrawal S	2012	India	200	300	IL-1α (-889C/T)	****	*	*
						IL-1α (+4845G/T)			
						IL-1β (-1903C/T)			
						IL-1β (-5887C/T)			
						IL-1β (+3593C/T)			
						IL-1β (-511T/C)			
21	Ma X	2012	China	162	156	IL-6 (-634C/G)	****	*	*
22	Messaoudi S	2012	Tunisia	235	235	IL-18 (-119A/C)	****	*	*
						IL-18 (-137G/C)			
						IL-18 (-105G/A)			
						IL-18 (-656C/A)			
23	Alkhuriji AF	2013	Kingdom of Saudi Arabia	65	65	IL-6 (- 634C/G)	****	*	*
						IL-10 (-592C/A)			
24	Drozdzik M	2013	Germany & Poland	157	158	IL-6 (-174G/C)	****	*	*
25	Parveen F	2013	India	200	300	IL-6 (-174 G/C)	****	*	*
						IL-4 (-590 C/T)			
						IL-10 (-819 T/C)			
						IL-10 (-1082 A/G)			
						IL-10 (-592 C/A)			
26	Bahadori M	2014	Iran	85	104	IL-6 (-174 G/C)	****	*	*
						IL-10 (-592 C/A)			
						IL-10 (-819 T/C)			
						IL-10 (-1082 A/G)			
						IL-17 (-197 A/G)			
27	Camil L	2014	Romania	69	64	IL-6 (-174G/C)	****	*	*
						IL-10 (-592C/A)			
						IL-10 (-819C/T)			
						IL-10 (-1082A/G)			
28	Demirturk F	2014	Turkey	113	113	IL-6 (-572 G/C)	****	*	*
						IL-6 (-597 G/A)			
						IL-6 (-1363 G/T)			
						IL-6 (-174 G/C)			
						IL-6 (-2954 G/C)			
29	Kim JO	2014	South Korea	385	232	IL-1β (-511C/T)	****	*	*
						IL-10 (-1082G/A)			
30	Najafi S	2014	Iranian	85	85	IL-17	****	*	*
31	Yue J	2015	China	484	486	IL-18 (-105G/A)	****	*	*
						IL-18 (-137G/C)			
						IL-18 (-656C/A)			

The quality assessment of the included articles is presented in Table 1. All of the articles are case-control studies. The observation studies scored using the Newcastle-Ottawa scale all received scores of six, except for one[19] (the definition of the experimental group was not clear enough, but after assessment by all authors, we decided to include this article).

### IL-1β polymorphism

**Fig 2:**
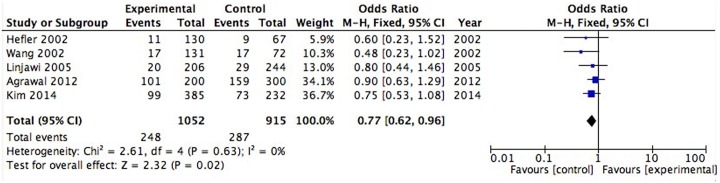
Association between the IL-1β (-511 C/T) polymorphism and RPL under a recessive genetic model.

The most common studied variant in IL-1β was the -511C/T polymorphism. The five included studies had a total of 1052 patients and 915 controls for analysis[4, 20–23]. Calculation of the ORs and 95% CIs for the fixed effects model showed a significant association under a recessive genetic model of IL-1β (-511C/T) polymorphism (P = 0.02, OR = 0.77, 95% CI [0.62,0.96] Fig 2) and no significant association under a dominant genetic model (P = 0.59, OR = 0.87, 95% CI [0.53,1.43], S1 Fig). Three studies included 348 patients and 401 controls in the analysis of IL-1β (+3594C/T)[1, 19, 22]. The ORs and 95% CIs for the fixed model indicated that the presence of the TT genotype at +3593 did not differ significantly in the patients with RPL and the control group in the dominant or recessive model (P = 0.15, OR = 1.25, 95% CI [0.92,1.68] S2A Fig; P = 0.97, OR = 0.99, 95% CI [0.57,1.73] S2B Fig). No significant heterogeneity was observed in these meta-analyses except for the IL-1β (-511C/T) dominant model (which will be explained in the discussion). In addition, one study reported that IL-1β (-31C/T) was also associated with RPL[4], but IL-1β (-1903C/T) and IL-1β (-5887C/T) were not[22].

### IL-6 polymorphism

**Fig 3:**
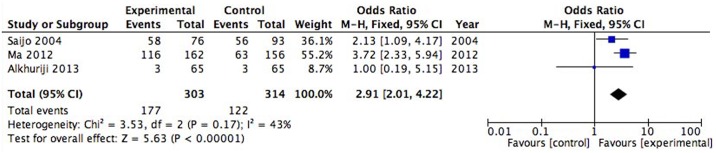
Association between the IL-6 (-634C/G) polymorphism and RPL under a recessive genetic model.

Twelve studies with a total 1000 patients and 1192 controls investigating the IL-6 (-174G/C) polymorphism were included in the analysis[3, 5, 14, 24–28, 31, 32]. The ORs and 95% CIs for the random effects model showed no difference between the RPL and control groups under the dominant and recessive models (P = 0.07, OR = 1.49, 95% CI [0.97,2.28] S3A Fig; P = 0.20, OR = 1.23, 95% CI [0.90,1.67] S3B Fig). Three articles with 303 patients and 314 controls investigating the IL-6 (-634C/G) polymorphism were included[27, 29, 30]. The meta-analysis indicated that it was a higher risk of RPL under the recessive model (P<0.001, OR = 2.91, 95% CI [2.01,4.22] Fig 3). No significant heterogeneity was observed in these meta-analyses. IL-6 (-572 G/C, -597 G/A, -1363 G/T) were not associated with RPL in one study, but there was a slightly higher appearance of heterozygosity in patients with RPL at the IL-6 (-2954G/C) locus (P = 0.033)[32].

### IL-10 polymorphism

**Fig 4:**
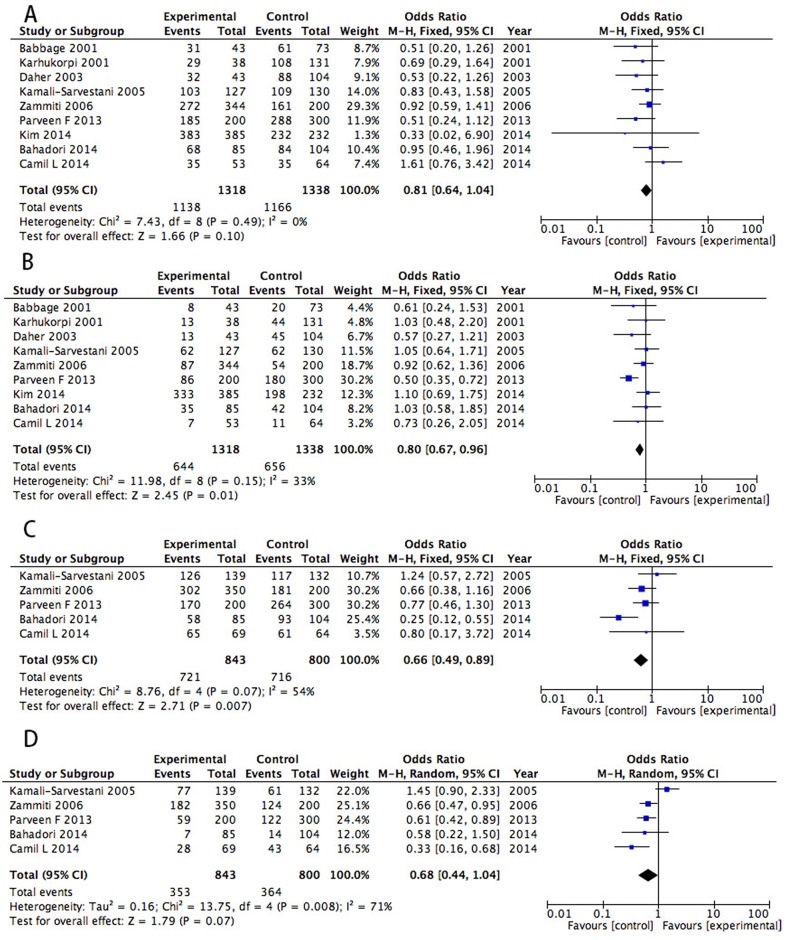
Association between the IL-10 (-1082G/A) (A, B) and IL-10 (–819 T/C) (C, D) polymorphisms and RPL under a dominant and recessive genetic models.

Nine studies with 1318 patients and 1338 controls investigating the relevance of the IL-10 (-1082G/A) polymorphism were included in the analysis[3, 14, 23, 24, 31, 33–36]. No association was observed in the IL-10 (-1082G/A) polymorphism under the dominant model (P = 0.10, OR = 0.81, 95% CI [0.64,1.04] Fig 4A), but there was a significant difference under the recessive model (P = 0.01, OR = 0.80, 95% CI [0.67,0.96] Fig 4B). Five articles with a total of 843 patients with RPL and 800 controls were included in the analysis[3, 14, 31, 35, 36]. Analysis of the ORs and 95% CIs for the random effects model showed a remarkable association between RPL and IL-10 (–819) genotype under the dominant, but not recessive, model (dominant: P = 0.007, OR = 0.66, 95% CI [0.49,0.89], Fig 4C; recessive: P = 0.07, OR = 0.68, 95% CI [0.44,1.04], Fig 4D). Seven studies with 951 patients and 915 controls were included in analysis for the IL-10 (-592C/A) polymorphism[3, 9, 14, 30, 31, 35, 36]. There was no significant difference with in the IL-10 (-592C/A) polymorphism in the RPL and control groups (dominant: P = 0.06, OR = 0.78, 95% CI [0.61,1.01] S4A Fig; recessive: P = 0.19, OR = 0.88, 95% CI [0.72,1.07] S4B Fig). No significant heterogeneity was observed in the meta-analysis. One study reported increased RPL risk with the IL-10 (-536A/G) polymorphism[42].

### IL-18 polymorphism

**Fig 5:**
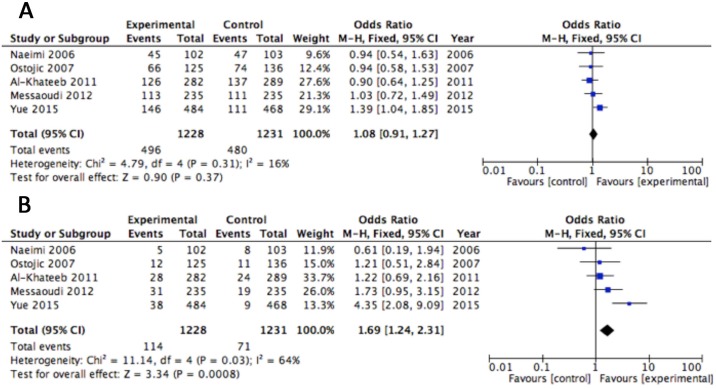
Association between the IL-18 (-137G/C) polymorphism and RPL under dominant and recessive genetic models.

**Fig 6:**
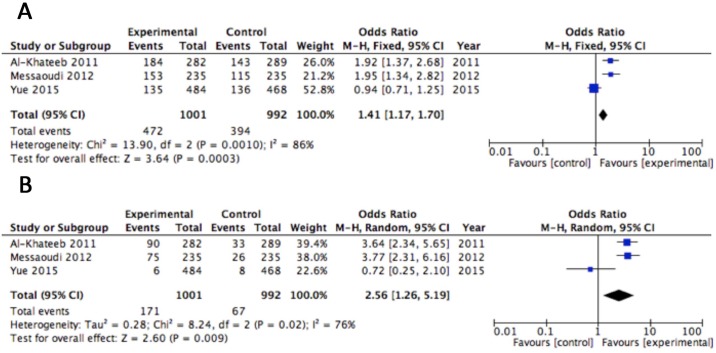
Association between the IL-18 (-105G/A) polymorphism and RPL under dominant and recessive genetic models.

Five studies with 1228 patients with RPL and 1231 controls were included in the meta-analysis[9, 38–41]. The fixed effect result indicated that the IL-18 (-137G/C) polymorphism was not associated with RPL under the dominant genetic model (P = 0.37, OR = 1.08, 95% CI [0.91,1.27], Fig 5A) but was associated under the recessive genetic model (P<0.01, OR = 1.69, 95% CI [1.24,2.31] Fig 5B). Two articles in the analysis investigated the association between RPL and the IL-18 (-607C/A) polymorphism in 227 patients with RPL and 240 controls[38, 39]. This polymorphism was not associated with RPL under either model (dominant: P = 0.31, OR = 0.82, 95% CI [0.56,1.21] S5A Fig; recessive: P = 0.17, OR = 0.73, 95% CI [0.47,1.14] S5B Fig). Two articles with 517 patients with RPL and 524 controls investigating the IL-18 (-119A/C) polymorphism were included[9, 40]. The effects analysis revealed no association between the IL-18 (-119A/C) polymorphism and RPL with either model (dominant: P = 0.50, OR = 0.92, 95% CI [0.72,1.17] S5C Fig; recessive: P = 0.69, 95% CI 1.09[0.71,1.68] S5D Fig). The heterogeneity among these studies was not significant. Three studies investigated the IL-18 (-656C/A) and IL-18 (-105G/A) polymorphisms with 1001 patients with RPL and 992 controls and were included in the analysis[9, 40, 41]. The P-values and 95% CIs demonstrated that the IL-18 (-656C/A) polymorphism is not associated with RPL (dominant: P = 0.09, OR = 1.74, 95% CI [0.92,3.28] S5E Fig; recessive: P = 0.12, OR = 1.66, 95% CI [0.88,3.16] S5F Fig), but IL-18 (-105G/A) shows a positive association (dominant: P = <0.01, OR = 1.41, 95% CI [1.17,1.70] Fig 6A; recessive: P<0.01, OR = 2.56, 95% CI [1.26,5.19] Fig 6B). However, this result should be interpreted cautiously because high heterogeneity was present. We attributed that the heterogeneity to ethnic differences.

#### Other interleukin polymorphisms

Only one study reported that the IL-4 (-590C/T) polymorphism was associated with RPL in northern Indian women under the dominant model[14]. One study reported that there was no statistically significant difference in the frequencies of IL-17 (-197G/A) polymorphisms between patients with RPL and the controls[31]. Another studied IL-17A rs2275913 and IL-17F rs763780 and indicated that rs763780 may be associated with RPL (P = 0.01, OR = 1.363, 95% CI [0.872–2.131])[8]. Rs2055979 and rs13143866 in IL-21 were also suspected to contribute to RPL risk in a 235-sample case-control study[10].

### The relationship between polymorphisms and gene promoters

**Fig 7:**
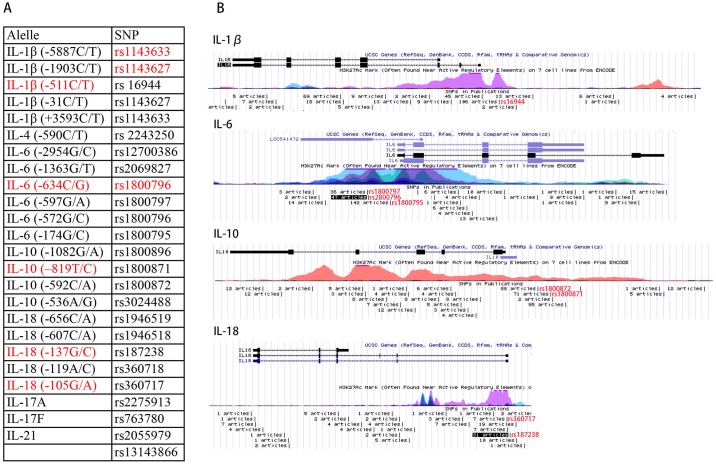
Corresponding SNP IDs and relative locations of these polymorphisms.

After meta-analysis, we found that five polymorphisms were related to RPL. To investigate their location and corresponding histone markers and sequence functions, we found their reference SNP ID Numbers (rs#) and located them with the UCSC Genome Browser Human Feb. 2009 (GRCh37/hg19). Based on seven H3K27Ac Mark Chip-seq results provided by UCSC, we found that almost all of the positive SNPs located in the H3K27Ac binding area, which indicated they are near regulatory elements (Fig 7). However, most negative polymorphisms were not located in H3K27Ac binding regions. Take IL-6 for example; the positive SNP IL-6 (-634C/G, rs1800796) is located in the area in which most Chip-seq data showed H3K27Ac binding. The negative SNP IL-6 (-174G/C, rs1800795), which is even closer to the transcription start site (TSS) than IL-6 (-634C/G), is located in a “valley” of these binding peaks.

## Discussion

In our systematic review, we analyzed seven interleukin genes and 21 polymorphisms in a total of 9401 patients. Twelve of the polymorphisms were included in the meta-analysis, and the others were only included in the review due to a limited number of reports. Our integrated results indicate that IL-1β (-511C/T), IL-6 (-634C/G), IL-10 (-1082G/A, –819T/C), IL-18 (-137G/C) and IL-18 (-105G/A) consistently associated with RPL. IL-17A rs2275913, IL-17F rs763780, IL-21rs2055979 and rs13143866, IL-1β (-31C/T), IL-4 (-590C/T), IL-6 (-2954G/C), and IL-10 (-536A/G) were reported on only once with respect to their association with RPL.

An increasing number of studies have demonstrated that a successful pregnancy depends on immune balance, including immunotolerance, the immune response and relative cytokines levels[43]. The corresponding immune cells that reside at the interface between the placenta and the uterus are subject to a superimposed layer of regulation by maternal immune cells[44]. These cells not only foster placental function and development but also reduce the possibility of the placenta attacking the fetus. Abnormal decidual leukocytes lead to RPL, intrauterine growth restriction, preeclampsia, etc. These leukocytes also secrete interleukins that act in placental immunoregulation[44].

Different interleukins are secreted by different immune cells and play distinct roles at different tissues. IL-1β is a pro-inflammatory cytokine that assists in B cell proliferation and maturation, natural killer (NK) cell activation and T cell stimulation[1]. The IL-1β (-511C/T) polymorphism leads to an increase in IL-1β production and the NK cell proportion of the lymphocytes population[20, 23]. These alterations are observed in women with RPL as a larger number of peripheral CD16+CD15+NK cells[45]. IL-6 takes part in trophoblast proliferation, differentiation and invasion and participates in follicle development and embryonic implantation[46, 47]. The IL-6 protein also plays a part in the initial spiral artery remodeling that requires vascular smooth muscle cell induction and morphological change. Reduced levels of IL-6 decrease trophoblast invasion and spiral artery remodeling[6]. The IL-6 (-634) promoter mutation reduces IL-6 transcription and expression directly, and this nucleotide alteration also provides a potential NF-1 transcription factor binding site, which plays a role in trophoblast function[47]. IL-10 takes part in the immunosuppressive response in congestion; high maternal IL-10 levels are associated with successful pregnancy and vice versa. The IL-10 (-819) polymorphism alters IL-10 secretion[48]. Other interleukins, such as IL-8, and IL-13, are also involved in pregnancy through trophoblast invasion and placenta formation. IL-18 is a pro-inflammatory cytokine induced by IFN-γ and aids in T cell polarization. Variation in the IL-18 promoter region influences IL-18 transcription and translation, and IL-18 protein expression is lower in patients with RPL[11]. Interleukins and corresponding immune cells work together to maintain the immune balance of mother and fetus.

All of these pregnancy-assisting functions of interleukins are based on normal interleukin expression and sequence and appropriate regulation, such as with DNA methylation and SNPs. In some situations, SNPs are located in the gene promoter; sometimes, they also appear within gene bodies and other non-coding regions. In these sensitive areas, even one base alteration will change relative protein binding and transcription levels[49]. These changes perhaps will become lethal to the sensitive immune balance between mother and fetus. Therefore, a SNP meta-analysis identifying the potential target out of a large population of SNPs and providing clinical evidence for relative gene promoter regulations is required. It can even provide clues about DNA folding, interactions, secondary structure formation and transcription complex function[50]. For example, IL-1β (-511C/T) is directly located on the promoter region of IL-1β in an area with a previous report of H3K27ac binding (often found near active regulatory elements) (Fig 7B). This demonstrates the potential significance of polymorphism to transcription factor binding.

Another issue in polymorphisms study is about inaccurate locus description. IL-1β (-5887C/T) and IL-1β (+3593C/T), IL-1β (-1903C/T) and IL-1β (-31C/T) share the same SNP ID, and after checking the Human Feb. 2009 (GRCh37/hg19) Assembly, we believe considered that IL-1β (+3593C/T) and IL-1β (-31C/T) seems to be more accurate descriptions for their location. And when we attempted to find the location of IL-6 (-634), we found that it shares the same accession number (rs1800796) with IL-6 (-572). Based on the variation in the versions of the human genome assembly, we recommend that clinicians use the SNP ID to detect polymorphisms in clinical cases and have provided a corresponding list of these locations in Fig 7A. The SNP ID will not change with genome assembly updates and is more convenient for aligning to Chip-seq results. We also completed a meta-analysis with the combined data for these loci and found still no difference (data not shown).

Ethnicity is also an important factor during SNP analysis. Different populations have different ancestors and migration histories. Linkage disequilibrium caused these SNPs to capture most genetic variations. DNA variation at promoters contributes to complex human traits by altering the spatial or temporal pattern of gene expression. To include more patients in the meta-analysis, we checked the allele frequency of each SNP in different populations in the NCBI dbSNP database (S1 Table). We found similar results for most of the allele frequencies in those populations included in our meta-analysis. In addition, the allele frequencies for specific ethnic populations in the NCBI database are similar to our data. Take IL-10 (-1082G/A) for example: Kim’s study found more than 99% of the people from Korea have allele A at this position, and the percentage in NCBI is 0.948. This result implies that IL-10 (-1082G/A) is unlikely to be a candidate for RPL in East Asian populations. However, our objective was to provide clues about the polymorphisms and possible gene expression mechanisms in RPL. Although there is some variation in different ethnic populations, it does not exclude these polymorphisms from the possibility of being high-risk loci for RPL.

Another source of potential bias arose from the definition of “normal controls”. Most studies defined controls of RPL as having no history of miscarriage or two previous live births. However, there was no clear definition of the control groups. Some papers defined controls as women with at least one successful pregnancy and without abortion; some used ethnically matched healthy controls; one article even used male blood samples[21]. Although the definitions were varied, at least they did not include the occurrence of spontaneous abortion. Maternal age was also considered to be one of the factors that affected the results of the meta-analysis; we only included studies corrected for maternal age by control group selection. This approach decreased the bias of maternal age in the meta-analysis.

Even though RPL is strictly defined as three or more consecutive miscarriages before 20 weeks, the standards used in the studies were different. Some studies included only uncertain miscarriages. Others defined RPL as more than two spontaneous abortions. After a discussion with all the authors, we included studies with this definition of RPL for the polymorphism analysis. One article selected patients attending a miscarriage clinic[19], and the description of miscarriage was different (spontaneous, unexplained, well-characterized). We also included this study after discussion among authors. We excluded other explained causes of miscarriage, such as uterine and luteal deficiency. We considered that polymorphism is an exploration of the frequency of genotypes among different populations.

Our review was the first attempt to calculate interleukin gene polymorphisms and to provide an estimate of the interleukin genes in women with RPL. Rigorous selection criteria were applied, and only those articles with reliable genetic detection methods and absolute definitions of RPL were taken into consideration. Two authors performed these analyses independently. To identify higher quality reports, we used the Newcastle-Ottawa Quality Assessment Scale to score each included paper (Table 1). Every study scored above five stars. We analyzed the combined effects under both dominant and recessive models and tested all the included studies under fixed or random effects of ORs. The combination of different studies not only elevated the statistical power but also provided a more precise estimation.

## Conclusion

Interleukin genes play important roles in the expression of these proteins and are further involved in the immune response to pregnancy. The present meta-analysis demonstrated consistent and remarkable associations between the IL-1β (-511C/T), IL-6 (-634C/G), IL-10 (-1082G/A, –819T/C), IL-18 (-137G/C) and IL-18 (-105G/A) polymorphisms. This review also showed that IL-17A rs2275913, IL-17F rs763780, IL-21 rs2055979 and rs13143866, IL-1β (-31C/T), IL-4 (-590C/T), IL-6 (-2954G/C), and IL-10 (-536A/G) correlate with RPL. Although these polymorphisms contain only one base change, our study added further evidence indicating that RPL is a polygenic disease, implying that these polymorphisms are potential markers for RPL.

## Supporting Information

S1 Checklist PRISMA 2009 checklist. (TIFF)

S2 Checklist Meta-analysis on Genetic Association Studies Checklist. (TIFF)

S1 Fig Association between the IL-1β (-511 C/T) polymorphism and RPL under a dominant genetic model. (TIF)

S2 Fig Association between the IL-1β (+3594C/T) polymorphism and RPL under dominant and recessive genetic models. (TIF)

S3 Fig Association between the IL-6 (-174G/C) polymorphism and RPL under dominant and recessive genetic models. (TIF)

S4 Fig Association between the IL-10 (-592C/A) polymorphism and RPL under dominant and recessive genetic models. (TIF)

S5 Fig Association between the IL-18 (-607C/A) (A, B), IL-18 (-119A/C) (C, D), and IL-18 (-656C/A) (E, F); polymorphism and RPL under dominant and recessive genetic models. (TIF)

S1 Table Allele frequency of included SNPs in different populations in the NCBI dbSNP database. (TIF)
