# Supplementary figures and images for: Association between Genetic Polymorphisms in Interleukin Genes and Recurrent Pregnancy Loss – A Systematic Review and Meta-Analysis
Source: PLoS One. 2017 Jan 19;12(1):e0169891. doi: 10.1371/journal.pone.0169891 (PMC5245830; doi:10.1371/journal.pone.0169891)

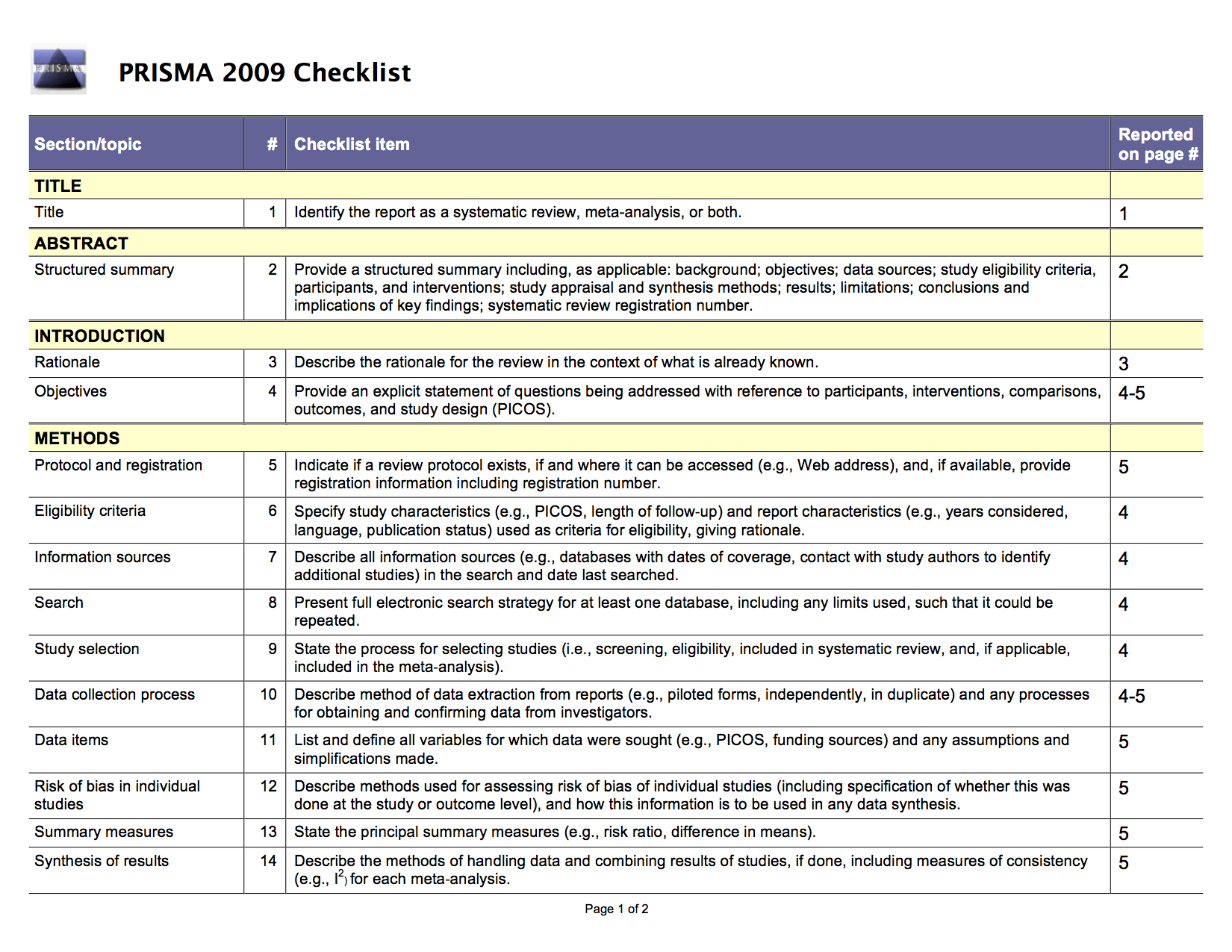

Supplement: S1 Checklist — (TIFF) [file pone.0169891.s001.tiff]

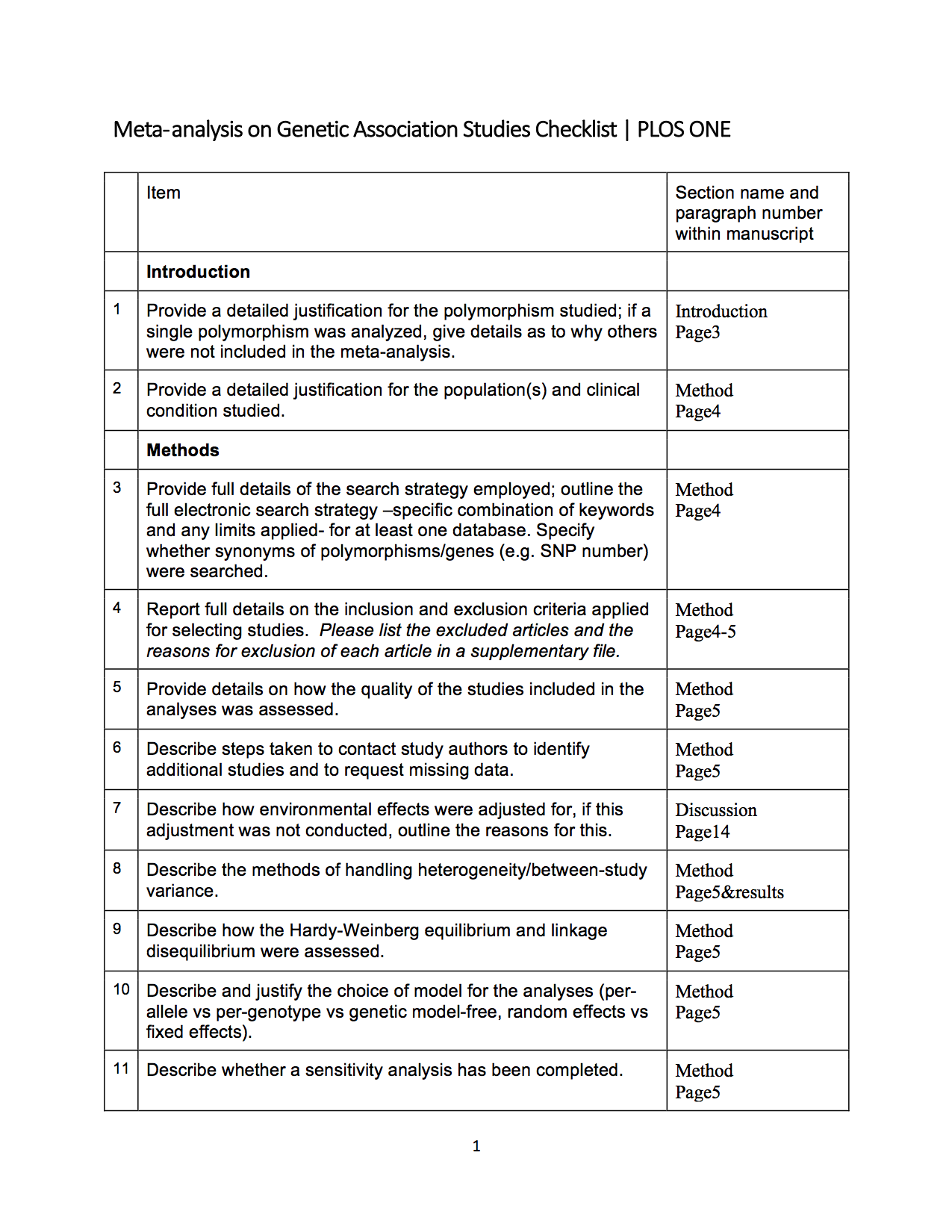

Supplement: S2 Checklist — (TIFF) [file pone.0169891.s002.tiff]

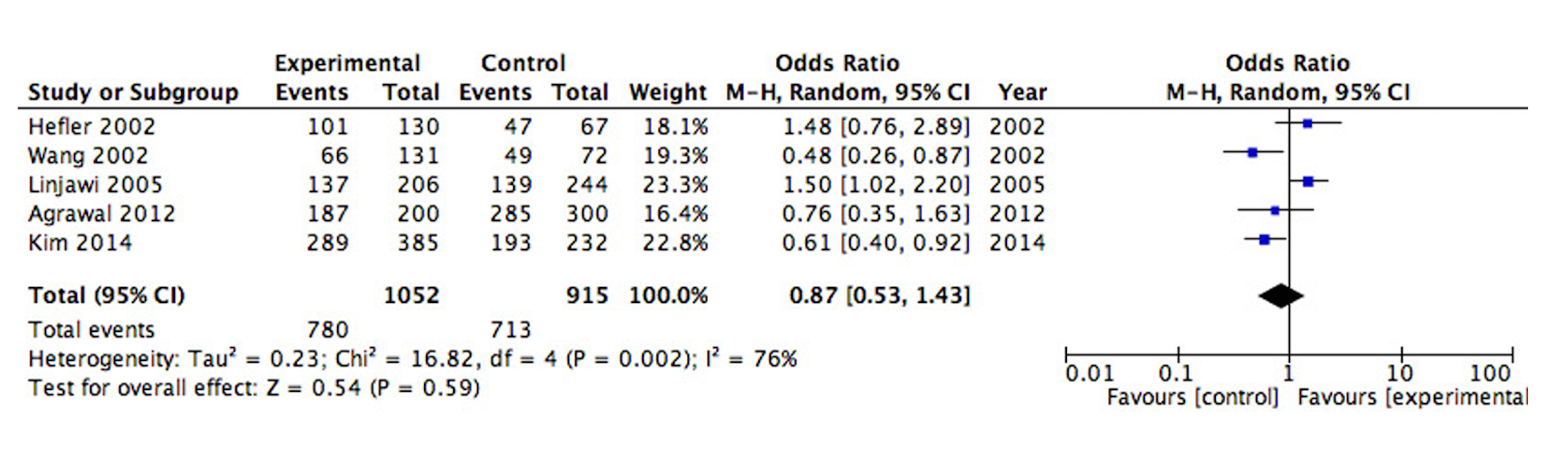

Supplement: S1 Fig — (TIF) [file pone.0169891.s003.tif]

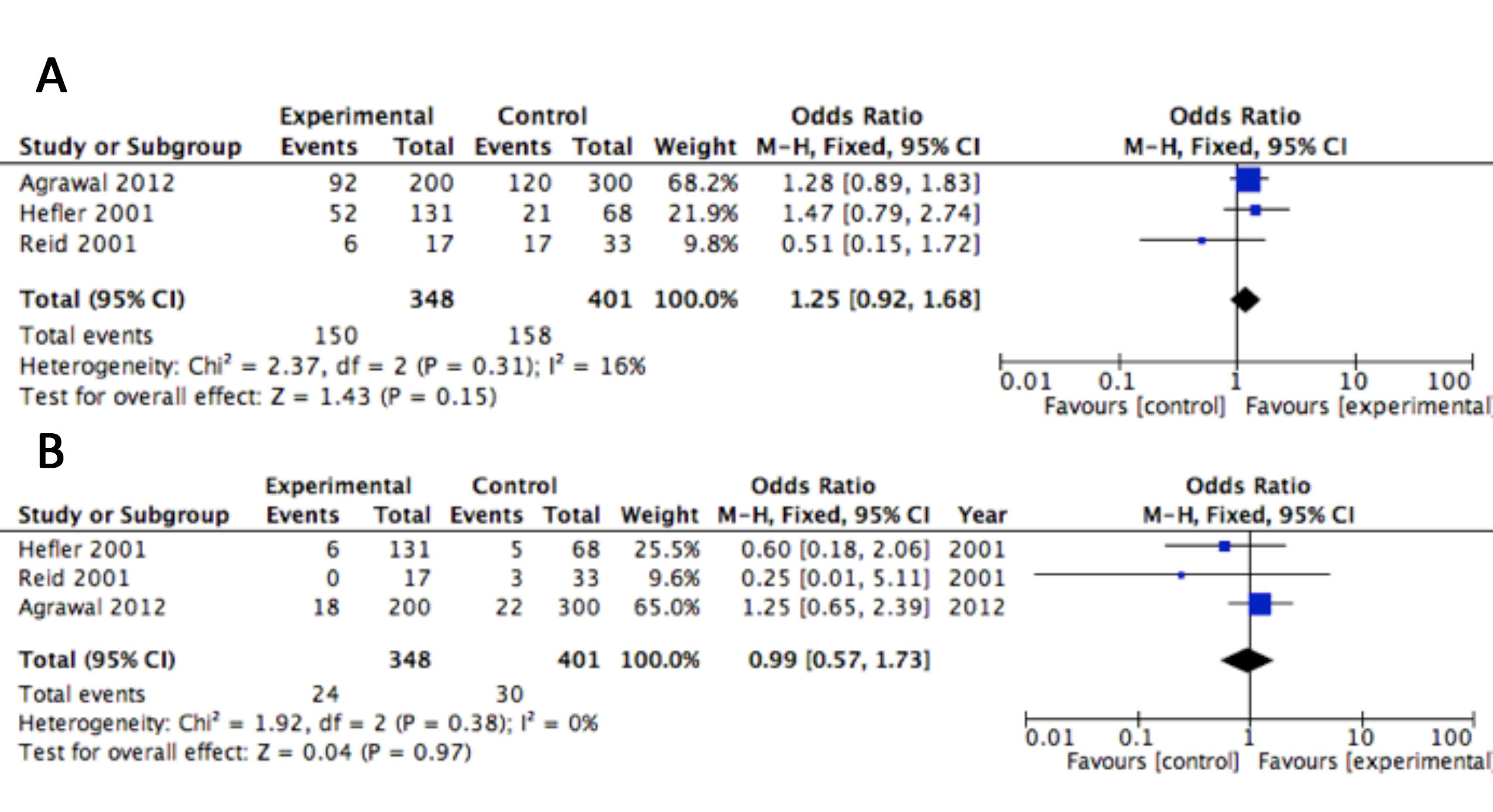

Supplement: S2 Fig — (TIF) [file pone.0169891.s004.tif]

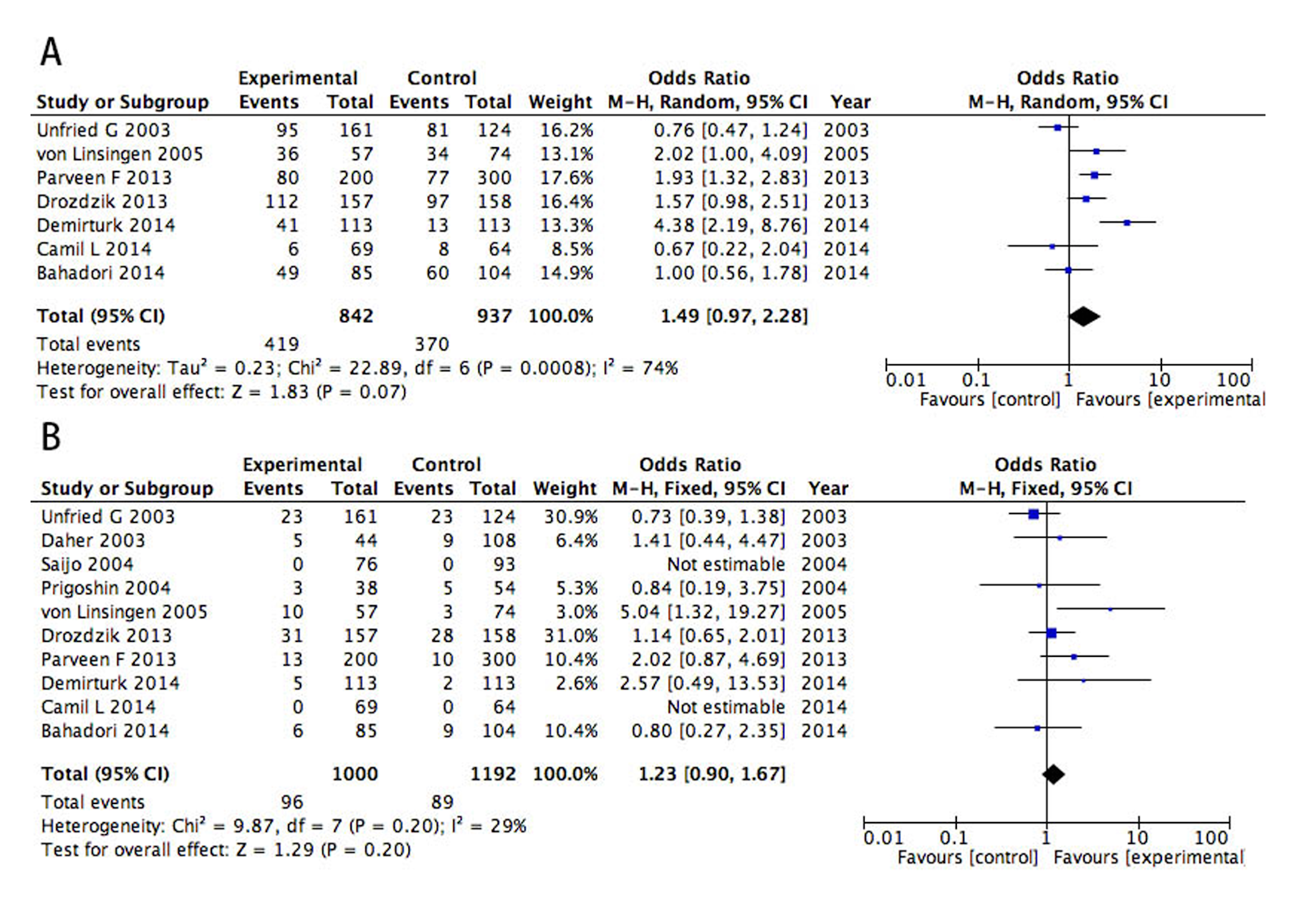

Supplement: S3 Fig — (TIF) [file pone.0169891.s005.tif]

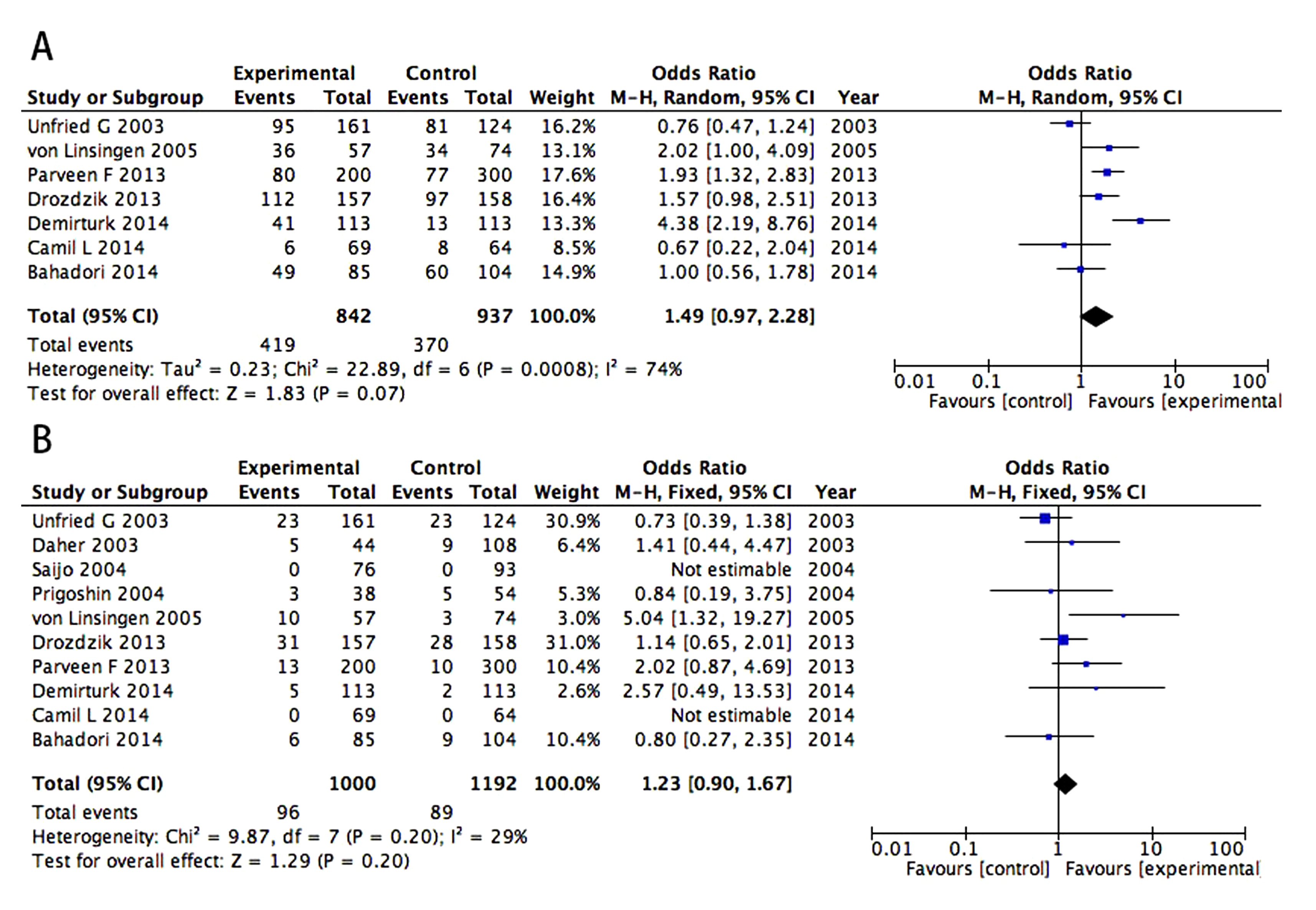

Supplement: S4 Fig — (TIF) [file pone.0169891.s006.tif]

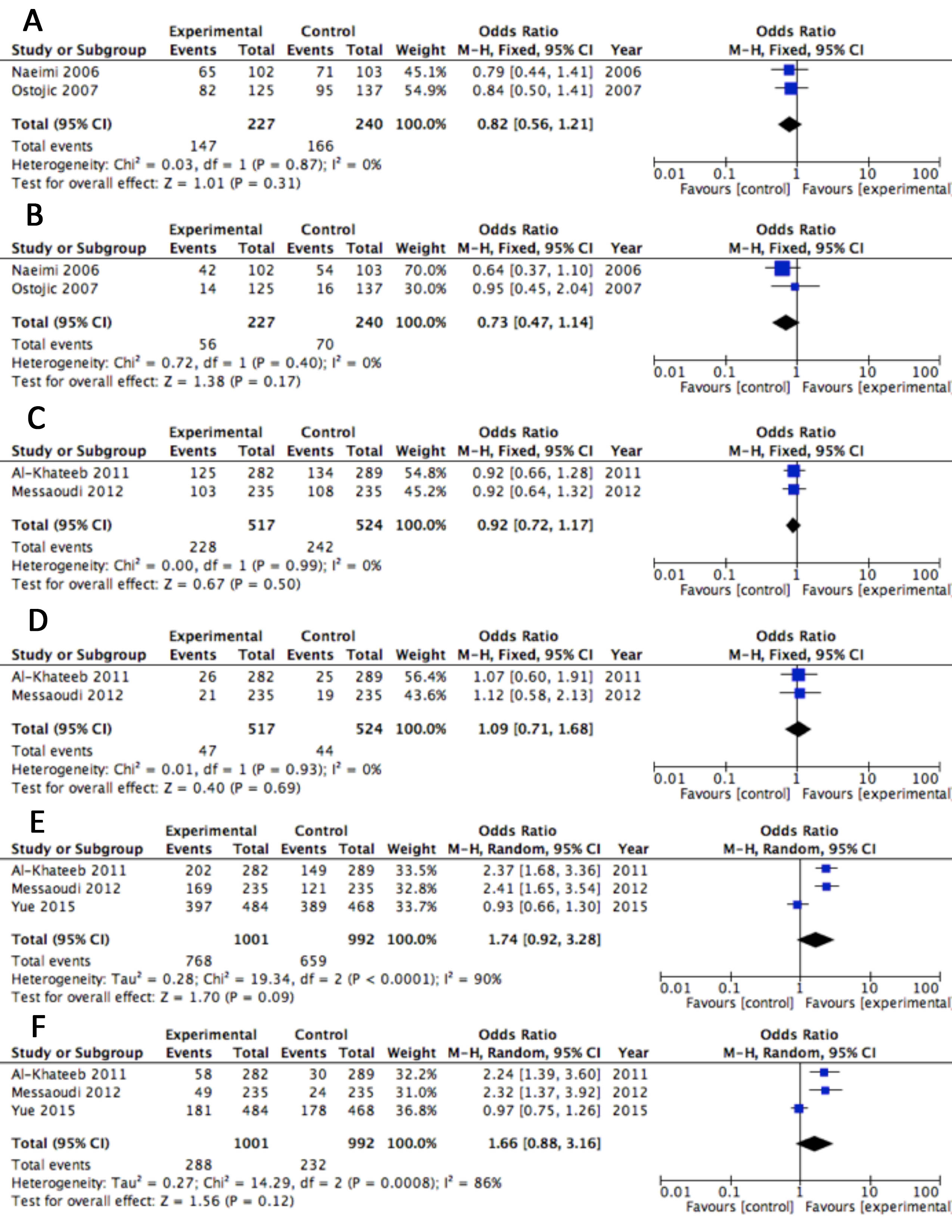

Supplement: S5 Fig — (TIF) [file pone.0169891.s007.tif]

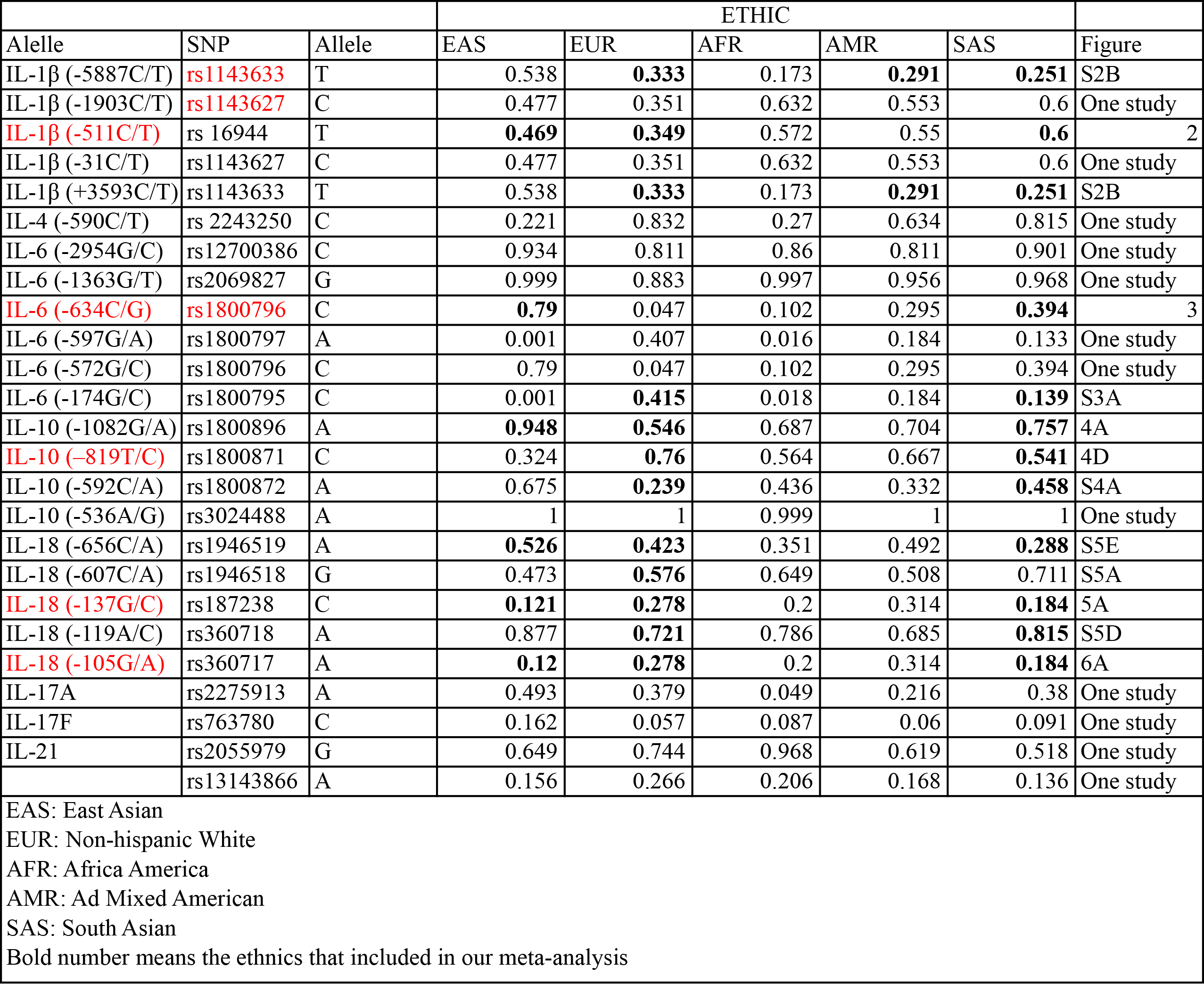

Supplement: S1 Table — (TIF) [file pone.0169891.s008.tif]
